# Effects of internet-based cognitive behavioral therapy on anxiety and depressive symptoms among patients with cardiovascular and cerebrovascular diseases: a systematic review and meta-analysis

**DOI:** 10.3389/fpsyt.2024.1433558

**Published:** 2025-01-16

**Authors:** Shuangyu Wang, Lishuo Gao, Congyu Wang, Jinbing Bai, Mengshuang Shen, Xuejie Zhao, Mei Lin

**Affiliations:** ^1^ Department of Cardiology, Tianjin Medical University General Hospital, Tianjin, China; ^2^ School of Nursing, Tianjin Medical University, Tianjin, China; ^3^ Department of Graduate, Tianjin Medical University, Tianjin, China; ^4^ Nell Hodgson Woodruff School of Nursing, Emory University, Atlanta, GA, United States; ^5^ Department of Nursing, Tianjin Medical University General Hospital, Tianjin, China

**Keywords:** cognitive behavioral therapy, cardiovascular diseases, anxiety disorders, depressive disorder, meta-analysis

## Abstract

**Background:**

This study aimed to evaluate the effectiveness of Internet-based Cognitive Behavioral Therapy (ICBT) in reducing anxiety and depressive symptoms among patients with cardiovascular diseases (CVDs) and to explore how intervention characteristics, such as module number and program duration, influence treatment outcomes.

**Methods:**

A systematic review and meta-analysis were conducted by searching eight databases, including PubMed, Embase, and Cochrane Library, for randomized controlled trials (RCTs) published up to December 2023. Studies involving adult CVD patients with anxiety or depressive symptoms who underwent ICBT interventions were included. Statistical analyses used random-effects models, with subgroup analyses performed to assess the impact of intervention format, module number, and program duration. Sensitivity and publication bias assessments ensured the robustness of the findings.

**Results:**

Eight RCTs with 1177 participants were included. ICBT significantly reduced depressive symptoms (SMD = -0.32, 95% CI [-0.56, -0.08], p < 0.015) and anxiety symptoms (SMD = -0.37, 95% CI [-0.68, -0.06], p < 0.001). Subgroup analysis indicated that self-guided ICBT was more effective than therapist-guided ICBT. Programs with fewer than eight modules were more effective for anxiety, while those with eight or more modules were more effective for depression. Shorter programs (< 9 weeks) were better for anxiety, whereas longer programs (≥ 9 weeks) were more effective for depression.

**Conclusions:**

ICBT is an effective intervention for managing anxiety and depression in CVD patients. Tailoring ICBT interventions based on symptom type, module number, and program duration can optimize outcomes. Future research should explore personalized, long-term strategies to enhance effectiveness and safety.

## 1 Introduction

Cardiovascular and cerebrovascular diseases (CVDs), encompassing ischemic heart disease, hypertension, heart failure, and stroke, represent one of the most pressing global health challenges of the 21st century (1, 2). These diseases not only constitute the leading cause of mortality worldwide, with approximately 17.5 million deaths annually (31% of total global deaths), but also impose an overwhelming burden on healthcare systems and society at large (3). Current projections indicate that CVD-related deaths will increase by 37% to reach 23.4 million by 2030 (4).

Within this disease spectrum, cerebrovascular disease, particularly stroke, presents a significant health burden. Stroke accounts for 10% of global deaths, with more than half of survivors requiring assistance with daily activities (5). Hypertension plays a crucial role in CVD progression by altering vascular structure and function (6).

Mental health complications, particularly depression and anxiety, frequently co-occur with CVDs and create a devastating cycle: psychological distress exacerbates cardiovascular symptoms, while deteriorating physical health further impacts mental well-being, significantly compromising patient outcomes. Depression is associated with a two-fold increase in mortality risk among CVD patients (7), while anxiety disorders double the risk of cardiac death compared to the general population (8). In patients with cerebrovascular disease, these psychological symptoms correlate with executive dysfunction and increased risk of subsequent strokes (9).

Cognitive behavioral therapy (CBT) has emerged as a gold-standard, evidence-based intervention for managing these psychological symptoms, demonstrating superior efficacy in reducing depression and anxiety symptoms compared to other psychological interventions. CBT’s structured approach specifically addresses the unique psychological challenges faced by CVD patients through systematic cognitive restructuring and behavioral activation. CBT helps patients identify and modify negative thought patterns through structured, time-limited psychotherapy sessions (10, 11). While traditional face-to-face CBT shows efficacy, its implementation faces numerous barriers, including limited accessibility, high costs, and therapist shortages (12).

Internet-based CBT (ICBT) represents a revolutionary advancement in psychological intervention delivery, gaining unprecedented prominence during the COVID-19 pandemic (13). This innovative approach combines the proven effectiveness of traditional CBT with the accessibility and scalability of digital health technologies, offering a potentially transformative solution for addressing the mental health needs of CVD patients. ICBT offers advantages over traditional CBT, including enhanced accessibility, privacy, and flexibility (14). Studies indicate that ICBT can match the effectiveness of traditional CBT while addressing many of its practical limitations (15).

While substantial evidence supports ICBT’s effectiveness in managing depression and anxiety among chronic disease patients (16, 17), there exists a critical knowledge gap regarding its specific application and effectiveness in CVD populations( (18, 19). Current literature shows inconsistent findings, varying methodological quality, and insufficient understanding of the optimal program characteristics for this unique patient group. Furthermore, the relationship between ICBT program characteristics (such as module content and duration) and treatment outcomes requires clarification.

This systematic review and meta-analysis aims to address these critical knowledge gaps by comprehensively examining the effectiveness of ICBT in managing anxiety and depression among CVD patients. By synthesizing existing evidence and analyzing program characteristics associated with optimal outcomes, this research will provide essential insights for developing targeted, evidence-based ICBT interventions for CVD patients and inform clinical implementation strategies.

## 2 Methods

The meta-analysis protocol was registered on the International Platform of Registered Systematic Review and Meta-analysis Protocols (INPLASY2023100040) on 10 October 2023.

### 2.1 PICOS statement

Population: Adult patients diagnosed with cardiovascular diseases (CVDs); Intervention: Internet-based cognitive behavioral therapy (ICBT); Comparator: Standard care, waitlist control, or other active treatments.

### 2.2 Search strategies

We searched the following databases from their inception to December 2023 for relevant RCTs, including The Cochrane Library, PubMed, Embase, Web of Science, PsycINFO, Chinese Biomedical (CBM) database, China National Knowledge Infrastructure (CNKI), and WanFang database. These included studies were searched using GoogleScholar. All the searches were performed using the following keywords without language restrictions, including “internet-based intervention”, “cognitive behavioral therapy”, “cardiovascular diseases”, and “cerebrovascular disorders”. Two reviewers screened the searched records by titles and abstracts and then reviewed the full text of eligible RCTs. The reference lists of identified studies were further searched to reduce the missing trials. A third reviewer was invited to help with the decision if the two reviewers had discrepancies in the inclusion of studies.

### 2.3 Eligibility criteria

#### 2.3.1 Studies and participants

Only RCTs were eligible. Patients with CVDs diagnosed with symptoms of anxiety or depression, and the RCTs reported the effects of the ICBT intervention on anxiety or depression were included, regardless of patients’ gender, age, ethnicity, types of cardiovascular disease, stage of cardiovascular disease, and current treatment.

#### 2.3.2 Interventions and controls

Interventions and controls: The included RCTs evaluated ICBT delivered in the following formats: Individual therapy, Group therapy, Self-guided programs, Therapist-guided programs, Combined formats (individual + group), ICBT programs of any frequency or duration were included, with treatment delivery via web platforms, mobile applications, or other internet-based channels. We defined ICBT as structured cognitive behavioral therapy delivered via internet-based platforms, which may include Synchronous (real-time) sessions, Asynchronous modules, Interactive exercises, Homework assignments, Progress monitoring, Automated feedback systems, Optional therapist guidance (email, chat, or video consultation).

#### 2.3.3 Outcomes

Anxiety and depression symptoms were assessed using validated measurement tools, including but not limited to: Hospital Anxiety and Depression Scale (HADS), Beck Depression Inventory (BDI), Patient Health Questionnaire-9 (PHQ-9), Generalized Anxiety Disorder-7 (GAD-7), Hamilton Rating Scales. Only studies using validated instruments with reported psychometric properties were included.

### 2.4 Exclusion criteria

Studies were excluded if they: Combined ICBT with face-to-face therapy, used non-internet-based delivery methods, included patients with severe psychiatric comorbidities, lacked clear ICBT protocol description, had insufficient outcome data, used non-validated measurement tools.

### 2.5 Screening and data extraction

Two trained reviewers administered a data collection form and subsequently extracted outcome data independently. The extracted data were then cross-referenced by these two reviewers and any inconsistencies were addressed through thoughtful discussion. In cases where data was not readily available, we made efforts to reach out to the authors of the study.

### 2.6 Risk of bias assessment

Our team of experts thoroughly analyzed the included studies, utilizing the esteemed Cochrane Handbook for Systematic Reviews to ensure the validity of the findings (20). When there were conflicting results between reviewers, our reviewers engaged in an in-depth discussion to make the decision. If a consensus cannot be reached, a third reviewer is involved to determine the results.

### 2.7 Data analysis

Statistical analyses were performed using STATA version 18.0. Effect sizes were calculated using random-effects models unless heterogeneity was low (I² < 50%, p > 0.1). Continuous variables were analyzed using standardized mean difference (SMD). 95% confidence intervals (CI) were calculated for each effect size. The I² test was used to verify the heterogeneity between studies, and sensitivity analysis was performed according to the single study deletion method to test the stability of the results. If the heterogeneity between studies was small (p > 0.1, I² < 50%), the high homogeneity of the research results can be considered, and the fixed effect model was used for analysis. When substantial heterogeneity (p ≤ 0.1, I² ≥ 50%) was observed, there may be heterogeneity between the results, and a random-effects model was used for our analysis (20).

Subgroup analysis was conducted to examine the outcome measures in this study. We utilized sensitivity analysis to eliminate studies that deviated substantially from the rest in terms of their approach or conclusions. By systematically removing each study and comparing the revised findings to the initial ones, we were able to reevaluate the data using a meta-analysis approach.

### 2.8 Reporting bias assessment

The reporting and publication biases of included studies were evaluated by visual inspection of the asymmetry of the funnel plot. Begg’s and Egger’s tests were used to test the publication bias when the number of the included studies was > 5 for a given outcome.

## 3 Results

### 3.1 Search results

**Figure 1:**
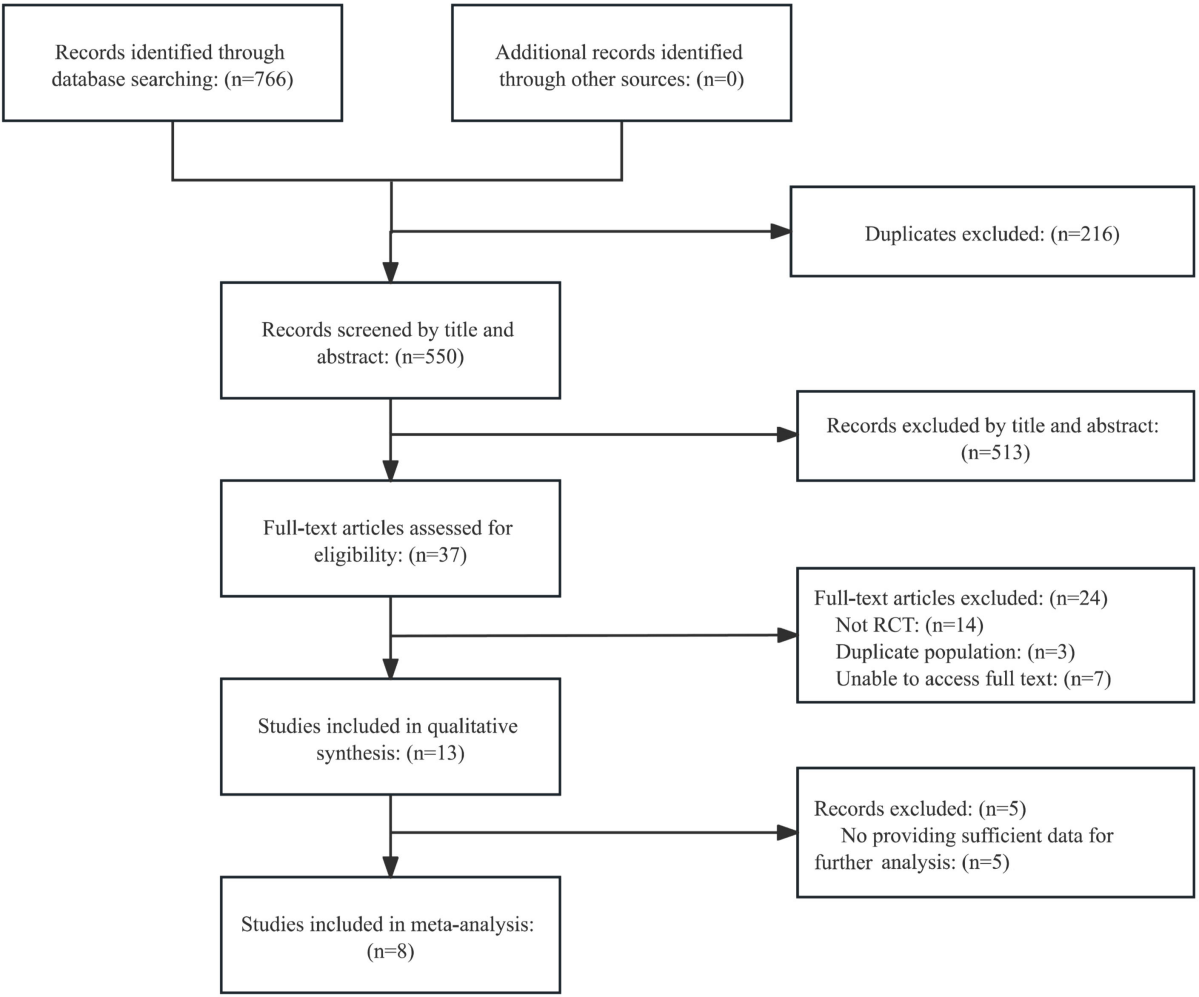
Flowchart of this study.

As shown in **Figure 1**, Database searches yielded 766 records: Cochrane Library (n=259), PubMed (n=95), Embase (n=180), Web of Science (n=95), PsycINFO (n=16), CBM (n=15), CNKI (n=62), and Wanfang (n=44). After removing 216 duplicates, 550 records remained for screening. Subsequently, upon an initial examination of the titles and abstracts, it was determined that 513 records did not fulfill the specified inclusion criteria and were excluded. When reading the full text, 24 records were deleted (14 non-RCTs, 3 duplicate populations, and 7 unable to access). In addition, 5 cases were excluded because useful data could not be obtained for further analysis. Finally, 8 studies were included in the meta-analysis.

### 3.2 Study characteristics

Eight articles were incorporated into this meta-analysis. **Table 1** provides an overview of the included studies, including various factors: subject demographics (location, sample size, age, gender, and type of CVDs), characteristics of the ICBT (form, number, and content of modules, duration, frequency, and program length), the control group, outcome measures, time points of data collection, and any reported adverse events.

**Table 1:** Studies included in the meta-analysis.

Author, year	Country	Sample	Age (mean ± SD)	Gender (female/male)	CVDs type	Intervention group: form, number of modules, duration, frequency, program length	Control group	Outcome indicator	Time points	Adverse events
Glozier, 2013 (21)	Australia	IG: 280 CG: 282	IG:57.5 ± 6.6 CG:58.4 ± 6.6	IG: 173/107 CG: 172/110	Not reported	ICBT (self-guided), 12 modules; 30-60 min/time, once a week, 12 weeks	Health Watch	PHQ-9; GAD-7	baseline; 12 weeks	None
Lundgren, 2016 (18)	Sweden	IG:25 CG:25	IG:63.6 ± 13.9 CG:62.3 ± 11.7	IG: 10/15 CG: 11/14	HF	ICBT (self-guided), 7 modules; unclear duration, once a week, 9 weeks	Discussion forum	PHQ-9; CAQ (total); CAQ (fear); CAQ (avoidance); CAQ (attention)	baseline; 9 weeks	Not reported
Simblett, 2017 (23)	England	IG:19 CG:9	IG:62.1 ± 11.4 CG:64.6 ± 8.1	IG:9/10 CG:1/8	Stroke	ICBT (self-guided), 8 modules; about 60 min/time, once a week, 8 weeks	cCRT	BDI-II; BAI	baseline; 12 weeks	Not reported
Norlund, 2018 (25)	Sweden	IG: 117 CG: 122	IG:58.4 ± 9.0 CG:60.8 ± 7.8	IG:44/73 CG:36/86	MI	ICBT (therapist-guided), 10 modules; duration ranged between 5 and 30 min, once a week, 14 weeks	TAU	HADS-T; HADS-A; HADS-D; MADRS-S; BADS-SF; CAQ	baseline; 14 weeks	Two patients in the iCBT group and 3 patients in the control or suicidal ideation at follow-up
Liang, 2018 (35)	China	IG:34 CG:33	IG:51.55 ± 6.10 CG:51.48 ± 12.89	IG:8/26 CG:14/19	Stroke	ICBT (therapist-guided), 6 modules; about 60 min/time, once a week, 8 weeks	Phone follow-up	PHQ-9; GAD-7	baseline; 4 weeks; 8 weeks	Not reported
Johansson, 2019 (26)	Sweden	IG:72 CG:72	IG: 61 ± 13 CG:64 ± 12	IG:25/47 CG:30/42	Mixed	ICBT (self-guided), 7 modules; unclear duration, once a week, 9 weeks	online discussion forum	PHQ-9; MADRS-S; GAD-7; CAQ (total); CAQ (attention); CAQ (avoidance); CAQ (fear)	baseline; 9 weeks	None
Schneider, 2020 (20)	Canada	IG:25 CG:28	IG:56.72 ± 11.90 CG:59.29 ± 6.93	IG:12/13 CG:19/9	Acute coronary event	ICBT (self-guided), 5 modules; unclear duration, 5 modules delivered online over 8 weeks, 8 weeks	Waiting-list Control	PHQ-9; GAD-7; CAQ (total); CAQ (attention); CAQ (avoidance); CAQ (fear); DASS-21(total); DASS-21 (depression); DASS-21(anxiety); DASS-21(stress)	baseline; 8 weeks	temporary increase in symptoms (n = 2) and discomfort in addressing symptoms (n = 1)
Bendig, 2021 (15)	Germany	IG18 CG:16	Total: 56.4 ± 10.2	Total: 12/22	Not reported	ICBT (therapist-guided), 8 modules; 45-60 minutes, once a week, 8 weeks	Waiting-list Control	PHQ-9; GAD-7	baseline; 8 weeks	None

IG, internet-based cognitive behavioral therapy group; CG, Control Group; CVDs, cardiovascular and cerebrovascular disease; HF, heart failure; MI, myocardial infarction; cCRT, computerized cognitive remediation therapy; TAU, Treatment as usual; PHQ-9, Patient Health Questionnaire-9; GAD-7, Generalized Anxiety Disorder-7; CAQ, Cardiac Anxiety Questionnaire; BDI-II, Beck Depression Inventory-II; BAI, Beck Anxiety Inventory; HADS-T, Hospital Anxiety and Depression Scale total score; HADS-A, Hospital Anxiety and Depression Scale anxiety subscale; HADS-D, Hospital Anxiety and Depression Scale depression subscale; MADRS-S, The Montgomery-Asberg Depression Rating Scale-Self Rated; DASS-21, depression anxiety stress scale.

### 3.3 Participants

The mean age of participants in all trials was 60.98 years (range = 50 - 70). Participants were predominantly male and only one trial (21) had > 50% women in both the intervention and control groups, and another trial (22) had > 50% women in the control group.

Regarding the type of CVDs, two trials (23, 24) involved patients with stroke, one (25)with myocardial infarction, one (22) with acute coronary event, and one (18) with heart failure. In addition, three trials did not identify the type of CVDs at the time of recruitment, and one (26) of these trials reported all types of CVDs in enrolled participants (atrial fibrillation or atrial flutter; coronary heart disease; heart failure), while the other two (15, 21) were not explicitly reported.

### 3.4 Intervention

The included trials were centered around the framework and material of the ICBT training program, adhering to the principles of CBT. Participants were able to conveniently complete all assessment measures online and send them in through a secure electronic platform. The interventions were facilitated either by experienced psychologists or through self-guided methods. The ICBT interventions were categorized into therapist-guided [n=3 (15, 24, 25)] and self-guided programs [n=5 (18, 21–23, 26)]. ICBT intervention modules of two trials (21, 25) were ≥ 10, and the remaining trials were < 10 but ≥ 5 modules. Most interventions ranged in duration from 30 to 60 minutes and were given once a week. The program length of the ICBT intervention ranged from 8 to 14 weeks.

### 3.5 Controls

The control group was a waiting group in one trial (22).Two trials used usual care as a control measure (24, 25). The other 5 trials used “active” control groups (15, 18, 21, 23, 26), meaning control measures with less feedback or less extension than the intervention group.

### 3.6 Risk of bias

**Figure 2:**
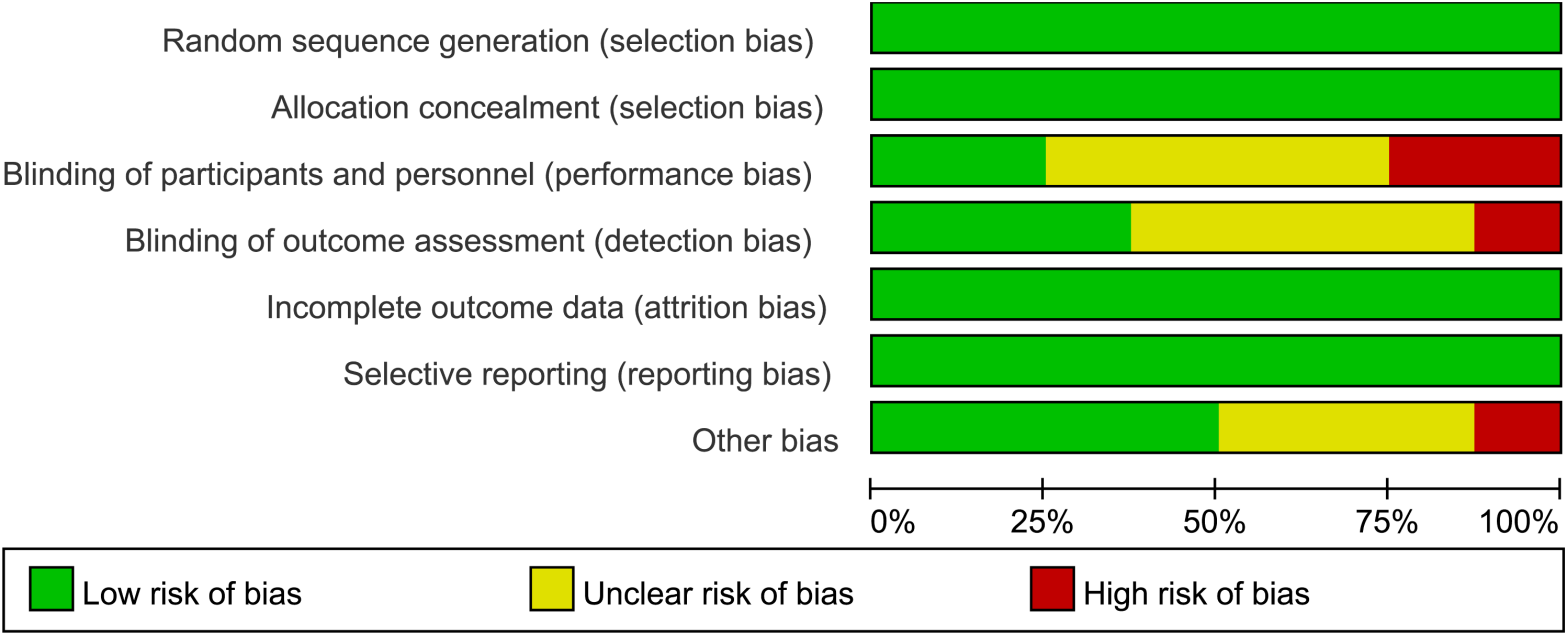
Risk of bias assessment using the Cochrane tool.

The risk of bias assessment by domain is shown in **Figure 2**. All studies had a low risk of random sequence generation, allocation concealment, and selective reporting. Details about participants and personnel blinding were provided in two studies (21, 24), while three trials (21, 24, 26) reported blinding of outcome assessment, so they were regarded low risk. In addition, three trials had an unclear risk of other bias (18, 23, 26), and one trial had a high risk of other bias (15) (**Figure 2**).

### 3.7 Analysis of overall effects

**Figure 3:**
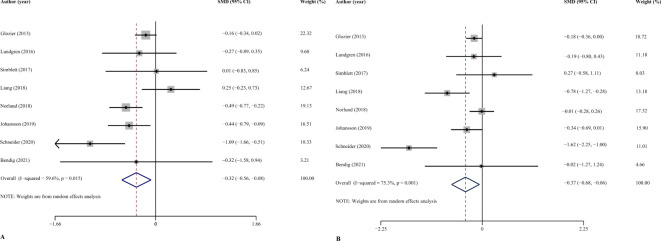
Overall effect of ICBT on depression **(A)** and anxiety **(B)**. 95%CI: 95% confidence interval.

All 8 studies (1177 patients) reported the effects of ICBT on the depressive and anxiety symptoms of CVD patients. The meta-analysis revealed a significant effect of ICBT in reducing depressive symptoms for patients with CVDs (SMD = -0.32, 95% CI [− 0.56, − 0.08], p < 0.015, I^2^ = 59.6%). Similarly, the ICBT was effective in reducing anxiety symptoms (SMD = -0.37, 95% CI [− 0.68, − 0.06], P < 0.001, I^2^ = 75.3%) (**Figure 3**).

**Figure 4:**
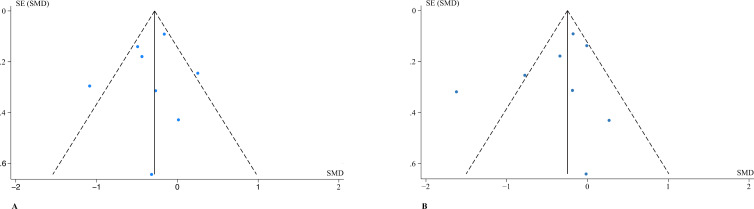
Funnel plot of comparison: overall effect of ICBT on depression **(A)** and anxiety **(B)**. SMD, standardized mean difference; SE, standard error.

Assessment of publication bias using Begg’s test (p=0.711) and Egger’s test (p=0.346) showed no significant publication bias for depression outcomes. Similar results were found for anxiety outcomes (Begg’s p=0.621; Egger’s p=0.405). Therefore, no significant publication bias was found. The two funnel plots did not reveal any significant asymmetry, supporting the stability of the results (**Figure 4**).

### 3.8 Subgroup and sensitivity analysis

#### 3.8.1 Form of intervention

We divided the forms of intervention into two groups: self-guided and therapist guided. The self-guided approach had a medium effect on depressive symptoms (SMD = -0.38, 95% CI [− 0.70, − 0.07], p = 0.035) but the effect of ICBT on anxiety (SMD = -0.42, 95% CI [− 0.86, 0.03], p < 0.01) is uncertain. The therapist-guided ICBT may have an effect, but the CI crossing zero renders it uncertain on depression (SMD = -0.18, 95% CI [− 0.76, 0.40], p = 0.031) and anxiety (SMD = -0.30, 95% CI [− 0.90, 0.30], p = 0.029) symptoms (**Tables 2**, **3**).

**Table 2:** Subgroup analyses of effects of ICBT on depression score changes in patients with CVDs.

Outcome type	K	ES	Sample size		I^2^	Random-effects analysis			
						SMD	95%CI		P
			IG	CG			L	U	
Form of intervention									
Self-guided	5	5	421	416	61.2%	-0.38	-0.70	-0.07	0.035
Therapist-guided	3	3	169	171	71.2%	-0.18	-0.76	0.40	0.031
Number of modules									
<8	4	4	156	158	76.1%	-0.37	-0.88	0.31	0.006
≧8	4	4	434	429	29.8%^*^	-0.25	-0.40	-0.11	0.234
Length of program									
<9 weeks	3	3	77	77	83.5%	-0.38	-1.37	0.60	0.002
≧9 weeks	5	5	513	510	22.7%^*^	-0.28	-0.41	-0.15	0.270

K, number of studies; ES, number of effect sizes; IG, Internet-based cognitive behavioral therapy group; CG, control group; SMD, standardized mean difference effect size; L, lower; U, upper; ^*^:applied fixed effect model.

**Table 3:** Subgroup analyses of effects of ICBT on anxiety score changes in patients with CVDs.

Outcome type	K	ES	Sample size		I^2^	Random-effects analysis			
						SMD	95%CI		P
			IG	CG			L	U	
Form of intervention									
Self-guided	5	5	421	416	80.8%	-0.42	-0.86	0.03	<0.001
Therapist-guided	3	3	169	171	71.9%	-0.30	-0.90	0.30	0.029
Number of modules									
<8	4	4	156	158	79.5%	-0.71	-1.27	-0.15	0.002
≧8	4	4	434	429	0.0%^*^	-0.11	-0.26	0.03	0.599
Length of program									
<9 weeks	3	3	77	77	71.3%	-0.93	-1.71	-0.15	0.031
≧9 weeks	5	5	513	510	0.0%^*^	-0.15	-0.28	-0.02	0.520

K, number of studies; ES, number of effect sizes; IG, Internet-based cognitive behavioral therapy group; CG, control group; SMD, standardized mean difference effect size; L, lower; U, upper; ^*^:applied fixed effect model.

#### 3.8.2 Number of modules

When the number of ICBT modules was < 8, the ICBT had insufficient evidence of an effect on depression (SMD =-0.37, 95% CI [− 0.88, 0.13], p = 0.006) symptoms, but a relatively large effect was observed on anxiety (SMD = -0.71, 95% CI [−1.27, -0.15], p = 0.002) in patients with CVDs. When the number of modules was ≥ 8, the ICBT had a small effect on relieving depression (SMD = -0.25, 95% CI [− 0.40, − 0.11], p = 0.234) symptoms, but the current data are insufficient to support the effect of ICBT on anxiety (SMD = -0.11, 95% CI [− 0.26, 0.02], p = 0.599) symptoms (**Tables 2**, **3**).

#### 3.8.3 Length of program

The ICBT intervention lasting for < 9 weeks had no sufficient evidence of effect on depression (SMD = -0.38, 95% CI [− 1.37, 0.60], p = 0.002) but showed a large effect on anxiety (SMD =-0.93, 95% CI [− 1.71, − 0.15], p = 0.031) symptoms in patients with CVDs. The ICBT intervention lasting for ≥ 9 weeks had a small effect on depression (SMD = -0.28, 95% CI [−0.41, -0.15], p = 0.270) symptoms but lack of statistical evidence for an effect on anxiety (SMD = -0.15, 95% CI [− 0.28, -0.02], p = 0.520) symptoms (**Tables 2**, **3**).

#### 3.8.4 Sensitivity analysis

**Figure 5:**
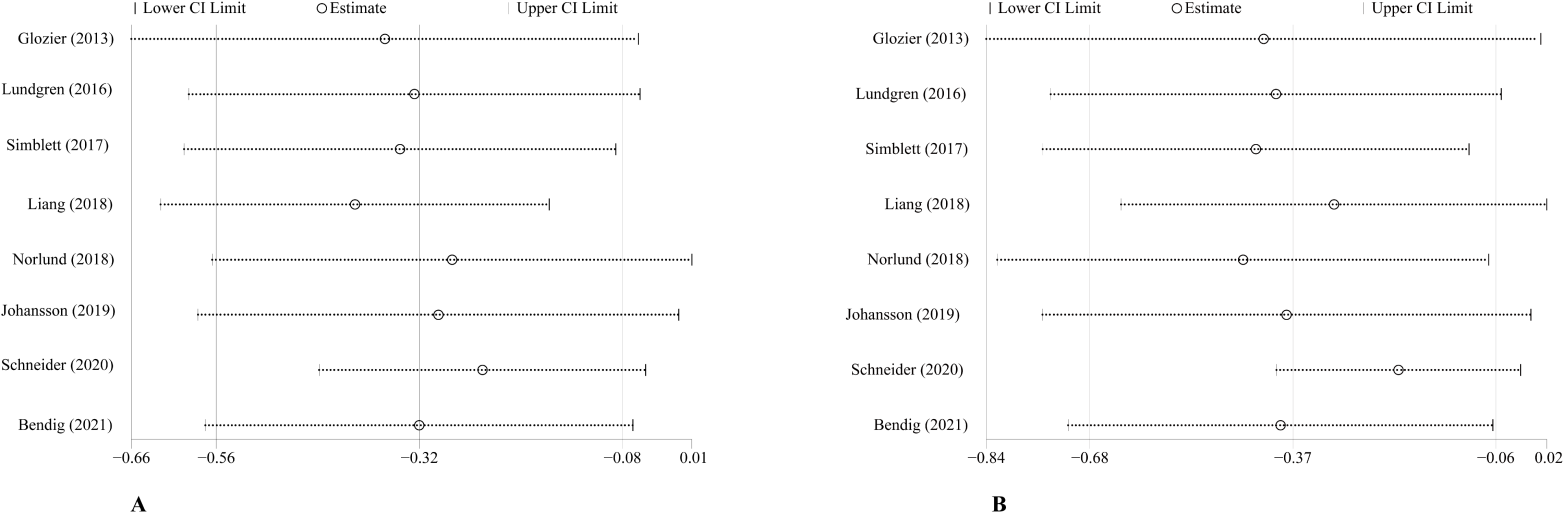
Sensitivity analysis of ICBT on depression **(A)** and anxiety **(B)**. CI, confidence interval.

After systematically removing the individual results of each study, the overall estimated effect remained largely unchanged. Therefore, no single study significantly influenced the overall conclusion. Although there were some variations among the studies, the results consistently demonstrated the efficacy of ICBT in alleviating symptoms of anxiety and depression (**Figure 5**).

#### 3.8.5 Adverse events

A total of five studies (15, 21, 22, 25, 26) reported adverse events associated with ICBT. One study (22) reported a temporary increase in symptoms after treatment in two cases and one reported feeling uncomfortable while dealing with symptoms. In addition, in the follow-up of another study (22), two participants from the ICBT group and three from the control group disclosed having suicidal thoughts. Other studies did not observe any negative occurrences (**Table 1**).

## 4 Discussion

This meta-analysis included 8 studies that evaluated how ICBT affected patients with CVDs regarding depressive or anxiety symptoms. According to these results, ICBT had a significant effect in diminishing symptoms of both depression and anxiety. This aligns with findings from two meta-analyses by Pang et al (27).and Bisby et al (28), which independently assessed whether internet-based psychological interventions were effective in reducing depression or anxiety. However, both meta-analyses included many different online psychological interventions. These meta-analyses included various psychological interventions, such as virtual mindfulness therapy and remote acceptance and commitment therapy, without specific focus on a single intervention type. Moreover, the samples were not limited to patients with CVDs. This heterogeneity in intervention types and patient populations limited the generalizability of conclusions. Our study aimed at patients with CVDs and only focused on ICBT. The subgroup analyses explored specific effects of different types and forms of intervention with varying lengths. Our findings supported which components of ICBT were most effective in relieving the symptoms of patients with CVDs.

We have classified intervention approaches into two categories: self-guided and therapist guided. We found that self-guided ICBT was more effective in easing symptoms of anxiety and depression among patients with CVDs than therapist-guided ICBT. This result is consistent with the results of previous work (29). This difference may be attributed to several reasons: first, self-guided ICBT enhances patient autonomy and flexibility so that patients can integrate the treatment into their daily routines with greater scheduling flexibility than regular live sessions or follow-up calls. This flexibility not only enhances treatment accessibility but also potentially increases therapeutic efficacy through increased patient autonomy in managing their treatment schedule. Second, self-guided ICBT can protect patient privacy and dignity (7). However, we acknowledge that therapist-guided ICBT may be more suitable for patients who need greater professional support and guidance. Treatment selection should be based on comprehensive evaluation of individual patient preferences, clinical needs, and specific circumstances. Current research on ICBT lacks studies that directly evaluate the relative efficacy of therapist-guided versus self-guided treatments in reducing anxiety and depressive symptoms among patients with CVDs. Future research needs to be conducted with meticulous attention to evaluate the advantages and disadvantages of each method of intervention. Furthermore, it would be highly beneficial to devise strategies for effectively integrating patients’ treatment preferences with various forms of intervention to attain optimal treatment results and enhance patients’ overall well-being.

The module number of ICBT interventions received particular attention in this meta-analysis, with studies involving between 5 and 12 different CBT modules. The analysis revealed that the efficacy of ICBT is significantly impacted by the number of modules. Specifically, when the number of modules is less than 8, ICBT is significantly effective in alleviating anxiety symptoms in patients with CVDs, possibly because the simplified number of modules facilitates more targeted therapeutic intervention, reduce the cognitive burden of patients, and make the treatment information easier to understand and practice, thereby reducing anxiety symptoms. When the number of modules reached eight or more, the ICBT improved depressive symptoms more significantly, which may reflect the need for more comprehensive intervention in the management of depressive symptoms, covering more psychosocial aspects, and helping patients to build a broader range of life management skills. These findings suggest that the number of modules and the depth of treatment should be adjusted according to the characteristics of symptoms when designing ICBT interventions. Page et al. tested the effects of ICBT with a different number of modules (16), which is inconsistent with the results of this study, suggesting additional research to determine the most effective interventions to address anxiety and depressive symptoms in patients with CVDs. Additionally, it would be beneficial to explore personalized treatment strategies to better meet the individual needs of each patient (30).

A significant correlation was identified between the duration of ICBT interventions and their effectiveness in alleviating anxiety and depression symptoms. Notably, the impact of intervention duration differed depending on the specific symptom targeted. Consistent with previous research (31, 32), shorter ICBT programs (less than nine weeks) were more effective in reducing anxiety symptoms in patients with CVDs. This may be due to the nature of anxiety disorders, which can respond rapidly to focused, short-term interventions that promote high levels of engagement (20). In contrast, addressing depressive symptoms often requires longer intervention periods to effectively modify the emotional, cognitive, and behavioral patterns associated with depression. Extended treatment durations allow patients to gain a deeper understanding of depression mechanisms, develop effective coping strategies, receive comprehensive professional skills training, and address underlying psychological issues. Therefore, when designing ICBT treatment plans, the duration should be tailored to the specific symptoms being addressed. It is essential to further investigate how varying treatment durations affect the reduction of anxiety and depression symptoms and to develop optimized treatment protocols for individuals with CVDs.

The analysis indicated no significant publication bias among the included studies, thereby enhancing the credibility of our findings. A comprehensive sensitivity analysis was performed to evaluate the influence of each individual study on the overall results. The removal of any single study did not alter the overall effect, suggesting that no single study disproportionately influenced the meta-analysis outcomes. Although some heterogeneity was observed across the study results, additional analyses confirmed that ICBT effectively reduced anxiety and depression symptoms in patients with CVDs.

Regarding adverse events, five studies in this study reported adverse events, including temporary exacerbation of symptoms after treatment, discomfort when dealing with symptoms, and a small number of patients reporting suicidal thoughts. These incidents remind us of the need to closely monitor patients when implementing ICBT, and the need to establish appropriate safety monitoring mechanisms and management measures to ensure patient safety. Incorporating all of our evidence, the evidence suggests that ICBT demonstrates significant therapeutic efficacy for addressing symptoms of anxiety and depression among individuals with CVD (33).however, due to the possibility of adverse events, enhanced monitoring protocols should be implemented when implementing ICBT interventions in the future to minimize potential negative effects and improve safety (34).

This study has several limitations. First, the quality assessment of the included literature may be subject to individual variability and subjectivity. Additionally, this meta-analysis did not include any ongoing studies, which may affect the comprehensiveness of the findings. The use of various scales to measure outcomes introduced heterogeneity, potentially reducing the accuracy of the results. Second, most included studies featured short trial durations and small sample sizes, which may limit the generalizability of the findings. Variations in ICBT types and intervention methods across studies could also influence the outcomes. Although ICBT interventions are generally considered safe, the incomplete reporting of adverse events hampers a thorough evaluation of their safety profile. Future research should undertake comprehensive examinations of different ICBT interventions and their effects on symptoms in patients with CVDs. Conducting long-term follow-up studies would also facilitate the assessment of the sustained efficacy of ICBT. Moreover, establishing a more robust system for documenting and reporting adverse events would enhance our understanding of ICBT applications in this population. Despite these limitations, our findings provide a foundation for future research aimed at improving the effectiveness and safety of ICBT protocols for individuals with CVDs.

## 5 Conclusion

This study demonstrated that Internet-based Cognitive Behavioral Therapy (ICBT) significantly reduces anxiety and depressive symptoms in patients with cardiovascular diseases (CVDs). Notably, self-guided ICBT was found to be more effective than therapist-guided ICBT in mitigating these symptoms. Additionally, ICBT interventions comprising fewer than eight modules were more effective in alleviating anxiety symptoms, whereas interventions with eight or more modules showed greater efficacy in addressing depressive symptoms. Regarding treatment duration, programs lasting less than nine weeks were more suitable for managing anxiety, while those extending to nine weeks or longer were more effective for depression. These results underscore the necessity of tailoring ICBT interventions to the specific symptoms and individual needs of patients with CVDs. Future research should focus on evaluating long-term and personalized ICBT strategies to further enhance the management of anxiety and depressive symptoms in this population.
