# Supplementary material for: Effects of internet-based cognitive behavioral therapy on anxiety and depressive symptoms among patients with cardiovascular and cerebrovascular diseases: a systematic review and meta-analysis
Source: Front Psychiatry. 2025 Jan 16;15:1433558. doi: 10.3389/fpsyt.2024.1433558 (PMC11779714; doi:10.3389/fpsyt.2024.1433558)
Supplement: Supplementary file 1 [file DataSheet1.zip › Supplementary Material Presentation/Supplementary Material-included articles.docx]

Bendig, E., Bauereiß, N., Buntrock, C., Habibović, M., Ebert, D.D., Baumeister, H., 2021. Lessons learned from an attempted randomized-controlled feasibility trial on "WIDeCAD" - An internet-based depression treatment for people living with coronary artery disease (CAD). Internet Interv 24, 100375.

Glozier, N., Christensen, H., Naismith, S., Cockayne, N., Donkin, L., Neal, B., Mackinnon, A., Hickie, I., 2013. Internet-delivered cognitive behavioural therapy for adults with mild to moderate depression and high cardiovascular disease risks: a randomised attention-controlled trial. PLoS One 8 (3), e59139.

Johansson, P., Westas, M., Andersson, G., Alehagen, U., Broström, A., Jaarsma, T., Mourad, G., Lundgren, J., 2019. An Internet-Based Cognitive Behavioral Therapy Program Adapted to Patients With Cardiovascular Disease and Depression: Randomized Controlled Trial. JMIR Mental Health 6 (10), e14648.

Lundgren, J.G., Dahlström, Ö., Andersson, G., Jaarsma, T., Köhler, A.K., Johansson, P., 2016. The Effect of Guided Web-Based Cognitive Behavioral Therapy on Patients With Depressive Symptoms and Heart Failure: A Pilot Randomized Controlled Trial. Journal of Medical Internet Research 18 (8).

Norlund, F., Wallin, E., Gustaf Olsson, E.M., Wallert, J., Burell, G., von Essen, L., Held, C., 2018. Internet-based cognitive behavioral therapy for symptoms of depression and anxiety among patients with a recent myocardial infarction: The U-CARE heart randomized controlled trial. Journal of Medical Internet Research 20 (3).

Rui, L., 2018. Study of Internet-based Cognitive Behavioral Therapy in Yong -middle Age Post Stroke Depression Patients.

Schneider, L.H., Hadjistavropoulos, H.D., Dear, B.F., Titov, N., 2020. Efficacy of internet-delivered cognitive behavioural therapy following an acute coronary event: A randomized controlled trial. Internet Interventions 21.

Simblett, S.K., Yates, M., Wagner, A.P., Watson, P., Gracey, F., Ring, H., Bateman, A., 2017. Computerized Cognitive Behavioral Therapy to Treat Emotional Distress After Stroke: A Feasibility Randomized Controlled Trial. JMIR Ment Health 4 (2), e16.
